# Chemical shift MR imaging in the lumbar vertebra: the effect of field strength, scanner vendors and flip angles in repeatability of signal intensity index measurement

**DOI:** 10.1186/s12880-016-0167-3

**Published:** 2016-11-25

**Authors:** Zebin Xiao, Jian Li, Chengqi Li, Yuyang Zhang, Dejun She, Dairong Cao

**Affiliations:** 1 Department of Radiology, First Affiliated Hospital of Fujian Medical University, 20 Cha-Zhong Road, Fuzhou, Fujian 350005 China; 2 Department of Radiology, Sanming Hospital of Integrated Traditional and Western, Sanming, Fujian 365000 China

**Keywords:** Chemical shift MRI, Repeatability, Bone marrow, In-phase and out-of-phase, Signal intensity index (SII)

## Abstract

**Background:**

To evaluate the reproducibility of signal intensity index (SII) measurements with MRI systems from different vendors and with different field strengths, and to test the effectiveness of flip angle.

**Methods:**

Thirty-two healthy volunteers (mean age 35.3 ± 9.3 years) were enrolled in this ethics committee-approved study. Chemical shift MR imaging was performed on 1.5- and 3.0-T MR systems from three vendors. Two independent observers measured SII values in five lumbar segments. Inter- and intraobserver agreement was assessed using the interclass correlation coefficients (ICCs). Differences of mean SII values between different field strengths and MR vendors as well as flip angles were compared by using repeated-measures analysis of variance. Differences of mean SII values between different flip angles were also compared by using paired-sample *t* test.

**Results:**

Inter- and intra-observer correlation coefficients showed good agreement (all ICC > 0.75) when measuring SII values at different MR systems (ICCs ranging from 0.896 to 0.983) and flip angles (ICCs ranging from 0.824 to 0.983). There were no significant differences in mean SII values measured by different MR vendors with different field strengths (all *p* > 0.05 ranging from 0.337 to 0.824). The differences in the mean SII between the four different flip angles were statistically significant (all *p* < 0.05 ranging from < 0.001 to 0.004) except the group of flip angle 50° versus 70° (*p* = 0.116).

**Conclusion:**

The SII measurement using chemical shift MR imaging may be comparable between different MR systems. Also high flip angles showed better stability to quantitate lumbar fat content.

## Background

Chemical shift magnetic resonance (MR) imaging (also known as in-phase and out-of-phase imaging or opposed-phased imaging) is a simple technique that takes advantage of the fact that water and lipid hydrogen protons in a single voxel show slightly different precession frequencies [1]. Based on phase differences in images acquired via different TEs, lipid and water signals are additive on in-phase images and subtracted on opposed-phase images [2]. This technique has been proved to be extremely useful for characterization of lesions and organs with fatty components and has gained widespread acceptance [3–6]. In clinical practice, it is widely used to diagnose lipid-poor adrenal adenomas [3, 7]. On the same bias, chemical shift MR imaging has been used to evaluate vertebral bone marrow fat content in osteoporosis or in distinguishing benign and malignant causes of vertebral bone morrow infiltration [2, 4, 7–16]. Furthermore, some investigators measured the signal intensity index (SII) value to avoid the problem of signal intensity variability produced by the reference tissue, and found that it appeared to be the most reliable method for differentiating adenomas from non-adenomas [7, 17]. However, there exists a major difference if measurements are performed at the adrenal gland or the bone marrow as reproducibility errors always have to be considered in relation to the variance that is expected in a population.

In spite of great interest in and enthusiasm about chemical shift MR imaging, there is a clear need for standardization of both the acquisition and the interpretation of chemical shift MR images to resolve current difficulties in comparing SII values from different studies or from different sites to enable validation of this quantitative parameter as a qualified biomarker in the context of multicenter studies.

SII measurements can be influenced by many factors such as chemical shift effect, susceptibility effect (i.e. T2* decay) and T1 relaxation, etc. [8, 18, 19]. Chemical shift effect occurs due to the slightly different precession frequency of water and fat. Susceptibility effect results from field inhomogeneity which is obvious at 3.0 Tesla or from the composition of the lesion itself [19]. As for the T1 relaxation, it depends on imaging parameters such as repetition time (TR) and flip angle [20]. Overall, whether SII values measured by chemical shift MR imaging can be compared across MR systems from different vendors and across field strengths remains an open question.

Thus, the aims of this study were to evaluate the reproducibility of SII values in the lumbar segments measured with MRI systems from different vendors and at different field strengths, and to test the effect of flip angle on assessing bone marrow fat content with respect to measurement of the signal intensity index (SII).

## Methods

This clinical study was approved by our Institutional Review Board (First Affiliated Hospital of Fujian Medical University) and written informed consent was obtained from all healthy volunteers enrolled in the study.

### Study population

In this present study, 35 healthy volunteers were included for chemical shift MR imaging of the lumbar spine between March 2015 and May 2015. The inclusion criteria for this study were as follows: (1) no history of trauma or surgery in the lumbar spine; (2) no history of acute or chronic pain in lower back; (3) no history of diseases which could change the signal intensity of lumbar marrow. The exclusion criteria was performed after interviewing the volunteers and reviewing their medical records, which included: (a) a history of or findings related to marrow diseases such as osteoporotic/traumatic fracture, traumatic, myeloma, osteosarcoma, lymphoma, spondylitis, etc.; (b) contraindications to MR imaging; (c) failure to complete the chemical shift imaging procedure for any reason; and (d) poor image quality insufficient for image analysis.

### MR imaging protocol

Data were acquired with 1.5 T MR systems (Vantage Altas, Toshiba Medical Systems, Otawara-shi, Japan) using phased-array spine coils with the following sequences: sagittal T1-weighted spin-echo sequence (500/10 [repetition time msec/echo time msec]), sagittal T2-weighted fast spin-echo sequence (4000/110 [repetition time msec/echo time msec]) and sagittal short inversion time inversion-recovery (STIR) fast spin-echo sequence (3500/65 [repetition time mse/echo time msec]). Chemical shift imaging data were acquired with 1.5 T MR systems from two vendors (Vantage Altas, Toshiba Medical Systems, Otawara-shi, Japan; Signa Twinspeed, GE Healthcare, Milwaukee, WI) and 3.0 T MR system from one vendor (Magneton Verio, Siemens Medical Solutions, Erlangen, Germany) using phased-array spine coils in one week. The sequence parameters on 1.5 T MR systems were as follows: sagittal out-of-phase (OP) (2.4/192 [repetition time mse/echo time msec]) fast spoiled gradient echo MR imaging was first scanned, followed by sagittal in-phase (IP) (4.8/192 [repetition time mse/echo time msec]) MR imaging. And the sequence parameters on 3.0 T MR system were as follows: sagittal in-phase (IP) (2.4/192 [repetition time mse/echo time msec]) MR imaging was first scanned, followed by out-of-phase (OP) (5.8/192 [repetition time mse/echo time msec]) fast spoiled gradient echo MR imaging. In addition, chemical shift MR imaging was performed on 3.0 T MR scanners with four flip angles (10, 30, 50, 70°), whereas only one flip angle at 70° was used in 1.5 T scanner. For all sagittal sequences, the field of view was 24 cm × 24 cm. The matrix was 256 × 256, and the section thickness was 4.0 mm, with a skip of 1.0 mm.

### Image analysis

All chemical shift MR imaging data were transferred to an independent workstation for evaluation. Two readers (Z.X. and Y.Z., radiologists with 2 and 4 years of clinical experience in musculoskeletal MR imaging, respectively) independently drew rectangular regions of interest (ROIs) with a specific size in the center of three representative sections of each vertebral body, including midsagittal sections and adjacent two sections. The ROI size was initially defined by two authors in consensus (D.C. and J.L., radiologists with 28 and 23 years of clinical experience in musculoskeletal MR imaging, respectively) in a test set of images. The ROI was defined based on the largest possible rectangular size measuring 1.0 cm × 1.5 cm for the respective sections, and size for each region was kept constant during placement of ROIs by using the function of copy and paste in our workstation. Thus, a total of 54 ROIs were collected for each volunteer (three sections per vertebral body, five vertebral body, three vendors with two field strength plus other three flip angles in three sections of one vertebral body in 3.0 T scanner, 3 × 5 × 3 + 3 × 3 × 1 = 54). Care was taken to exclude areas with obvious artifacts from the ROIs.

The signal intensity index (SII) was measured independently by two radiologists blinded for patient data (C.L. and D.S. with 6 and 4 years of clinical experience in musculoskeletal MR imaging, respectively). SII was defined as the percentage of signal change on the OP sequence compared to the IP sequence according to previously described equation [21, 22]: SII = (SI_IP_-SI_OP_)/SI_IP_ × 100%. SI_IP_ refers to mean signal intensity of the bone marrow on IP sequences and SI_OP_ refers to mean signal intensity of the bone marrow on OP sequences. For further analyses, the mean SII value of the two readers was calculated for each placed ROI.

### Statistical analysis

SII values were expressed as mean ± standard deviation (SD) and were tested first with the Kolmogorov-Smirnov test for normality and then with the Levene test for variance homogeneity.

Intra- and inter-observer agreement of SII measurements were assessed using intra- and interclass correlation coefficients (ICCs). An ICC greater than 0.75 was indicative of good agreement [23]. The mean differences, SD, and 95% limits of agreement (LOA) were calculated using the Bland-Altman method [24, 25]. Measurement repeatability was assessed by the Bland-Altman analysis in order to define the agreement between replicate measurements. The repeatability coefficient, which represents the threshold value below which the absolute differences between two measurements on the same subject is expected to lie for 95% of the measurement pairs, was assessed using the formula 1.96 × SD of the mean difference (dSD), and expressed as percentage of the mean SII value.

Differences of mean SII values between different field strengths and MR vendors as well as flip angles were compared by using repeated-measures analysis of variance. Next, differences of mean SII values between different flip angles were also compared by using paired-sample *t* test. The Bonferroni method was used to adjust for multiple comparisons when necessary.

All statistical calculations were performed using the commercially available software package (PASW, Version 18.0, SPSS Inc., Chicago, IL, USA). Differences were considered significant when *p* values were less than 0.05.

## Results

### Population demographics

Thirty-five volunteers were enrolled in this study. Three of them did not complete the study because of poor image quality (n = 2) or incomplete acquisition of all sequences (n = 1). Thirty-two volunteers successfully completed the imaging examinations (22 men and 10 women; mean age 35.3 ± 9.3 years; age range 21–50 years), and all the MR data sets were eligible for evaluation.

### Repeatability of SII measurements in different MR systems and flip angles

The SIIs measured in this study met the normal distribution (*p* = 0.193) and homogeneity of variance (*p* = 0.128). The intraobserver ICC calculated based on reader 1’s two measurements of SII values in 3.0 T Siemens system ranged from 0.896 to 0.972, with the 95% LOA ranging from (0.860, 0.923) to (0.962, 0.989) (Table 1). Interobserver agreement between reader 1’s first measurements and reader 2’s measurements was good for the three MR systems, with ICC (95% LOA) ranging from 0.916 (0.885, 0.938) to 0.983 (0.954, 0.975). The intra- and interobserver ICC results show good agreement between MR systems (all ICC > 0.75).

**Table 1:** Intra- and interobserver agreement for SII measurement with different field strengths and vendors (Flip angle = 70°)

Agreement	Siemens		GE		Toshiba	
	Reader 1/First time Reader 2/Second time		Reader 1/First time	Reader 2/Second time	Reader 1/First time Reader 2/Second time	
Intraobserver	0.935 (0.912, 0.952)	0.896 (0.860, 0.923)	0.961 (0.947, 0.973)	0.930 (0.905, 0.948)	0.972 (0.962, 0.989)	0.906 (0.874, 0.930)
Interobserver	0.983 (0.954, 0.995)	0.916 (0.885, 0.938)	0.954 (0.893, 0.971)	0.952 (0.935, 0.965)	0.976 (0.967, 0.982)	0.932 (0.878, 0.964)

SII, Signal intensity index; Note. —Data in parentheses are 95% LOA

As shown in Table 2, the intra- and inter-observer ICC (95% LOA) ranged from 0.824 (0.668, 0.907) to 0.983 (0.954, 0.995), showing good agreement in measuring SIIs in different flip angles (all ICC > 0.75).

**Table 2:** Intra- and interobserver agreement for SII measurement with different flip angles

Agreement	Flip angle = 10°		Flip angle = 30°		Flip angle = 50°		Flip angle = 70°	
	Reader 1/First time Reader 2/Second time		Reader 1/First time	Reader 2/Second time	Reader 1/First time Reader 2/Second time		Reader 1/First time Reader 2/Second time	
Intra-	0.824	0.881	0.961	0.913	0.926	0.936	0.935	0.896
observer	(0.668, 0.907)	(0.796, 0.941)	(0.944, 0.983)	(0.902, 0.941)	(0.854, 0.963)	(0.879, 0.964)	(0.912, 0.952)	(0.860, 0.923)
Inter-	0.858	0.891	0.969	0.912	0.923	0.971	0.983	0.916
observer	(0.671, 0.914)	(0.815, 0.943)	(0.923, 0.985)	(0.897, 0.937)	(0.852, 0.958)	(0.933, 0.989)	(0.954, 0.995)	(0.885, 0.938)

Note. —Data in parentheses are 95% LOA

*SII*, Signal intensity index

### Mutual agreement of mean SII measurements with different MR systems

The goal of this study was to assess agreement of mean SII values with different vendors at the same field strength (1.5 T Toshiba Vantage–1.5 T GE Signa), different field strengths and vendors (1.5 T GE Signa–3.0 T Siemens Verio; 1.5 T Toshiba Vantage-3.0 T Siemens Verio).

**Fig. 1:**
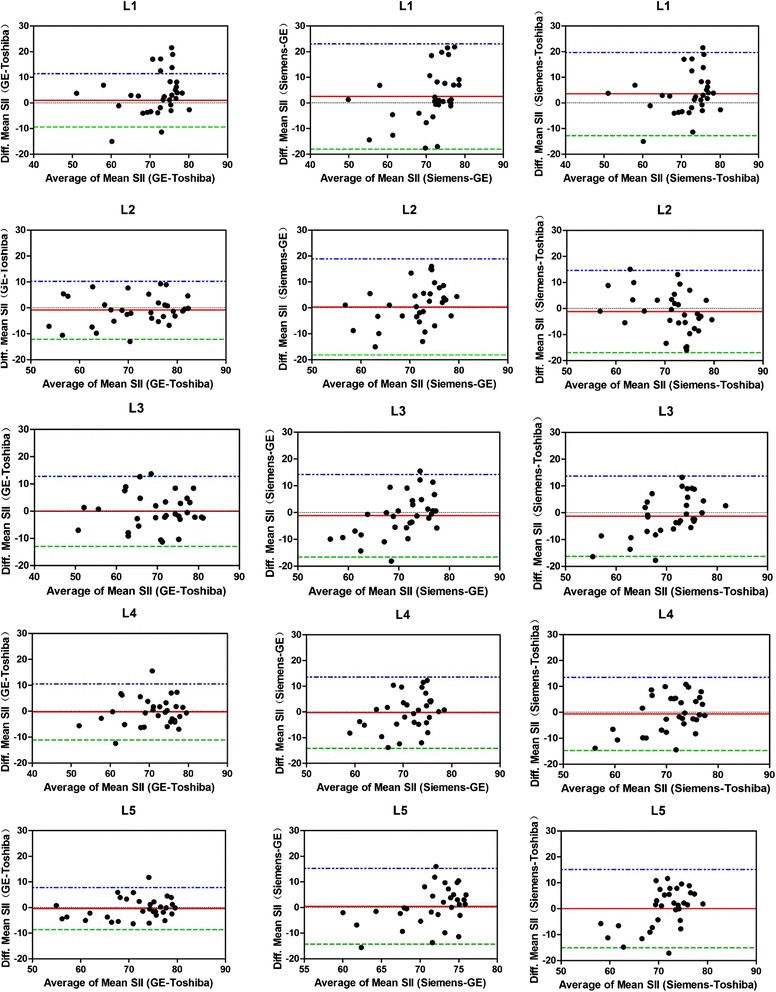
Mutual agreement of signal intensity index (SII) measured from different MR systems. The Bland-Altman plots show SII difference versus SII mean in different MR systems. For each lumbar segment, the mean SII difference (the red solid line) and the limits of agreements (lower limit: the green interrupted horizontal line; upper limit: the blue interrupted horizontal line) are shown. For reference, zero SII difference is shown as a black dotted line

**Fig. 2:**
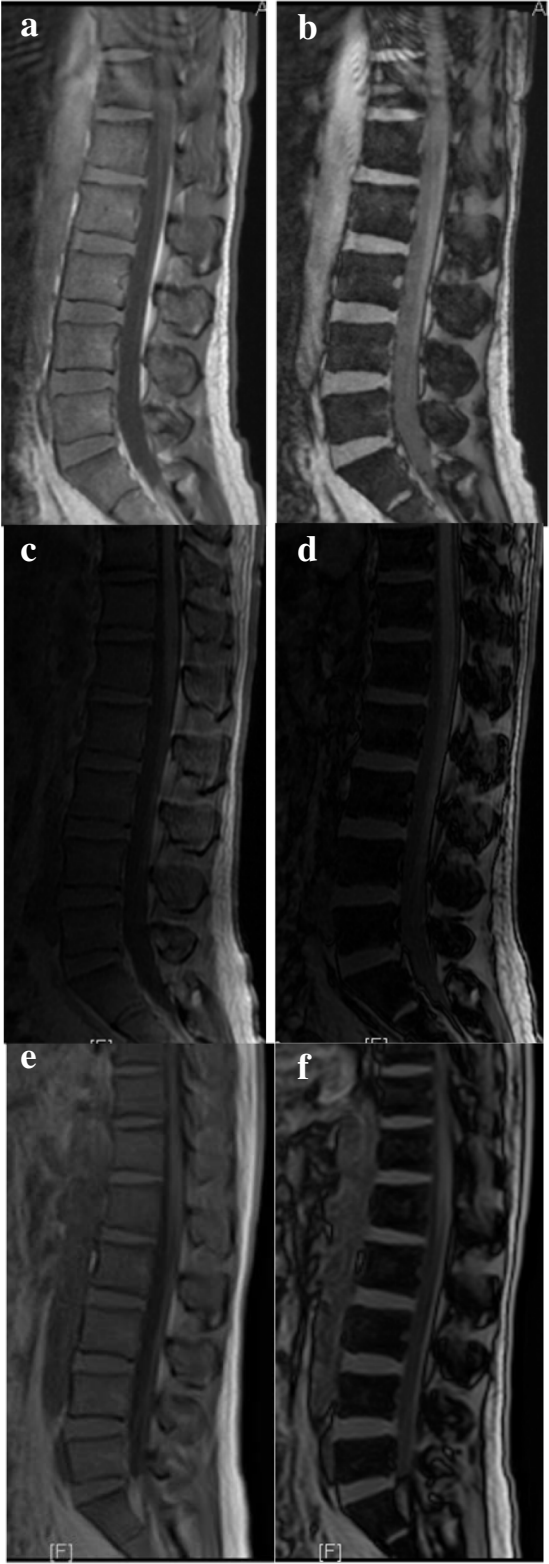
Representative images of chemical shift MR imaging in different MR systems. (**a**) in-phase image acquired at 3.0 T Siemens MR system; (**b**) out-of-phase image acquired at 3.0 T Siemens MR system; (**c**) in-phase image acquired at 1.5 T GE MR system; (**d**) out-of-phase image acquired at 1.5 T GE MR system; (**e**) in-phase image acquired at 1.5 T Toshiba MR system; (**f**) out-of-phase image acquired at 1.5 T Toshiba MR system

Overall, the agreement of above three conditions was desirable as the mean differences were very small (the mean differences ranged from −1. 324 to 3.462) and most of the data points lied within 95% LOA (Figs. 1, 2). As shown in Table 3, the bias was not systematic, but depended on the specific lumbar segments and on the different MR systems. For instance, there existed significant bias on the measurements of SII values in L1 with three different MR systems; some segments (for example L3) showed negligible bias in comparison within the same field strength (Toshiba-GE), but a little bias when combining 1.5 T and 3.0 T data (1.5 T GE–3.0 T Siemens and 1.5 T Toshiba–3.0 T Siemens).

**Table 3:** Reproducibility of Mean SII Measurements in Each of the Five Lumbar Segments with Three Vendors in Two Field Strengths

Lumbar Segment	1.5 T Toshiba– 1.5 T GE	1.5 T GE– 3.0 T Siemens	1.5 T Toshiba - 3.0 T Siemens
L1	1.002 (−9.384, 11.388)	2.460 (−18.067, 22.987)	3.462 (−12.770, 19.693)
L2	−0.906 (−12.100, 10.289)	−0.327 (−18.893, 18.239)	−1.233 (−17.012, 14.547)
L3	−0.094 (−12.940, 12.751)	−1.230 (−16.663, 14.203)	−1.324 (−16.319, 13.671)
L4	−0.303 (−11.095, 10.488)	−0.327 (−14.208, 13.554)	−0.631 (−14.787, 13.526)
L5	−0.452 (−8.683, 7.779)	0.456 (−14.329, 15.242)	0.004 (−15.133, 15.142)

Note. —Data in parentheses are 95% LOA

### Comparison of mean SII values with three vendors in Two field strengths

**Fig. 3:**
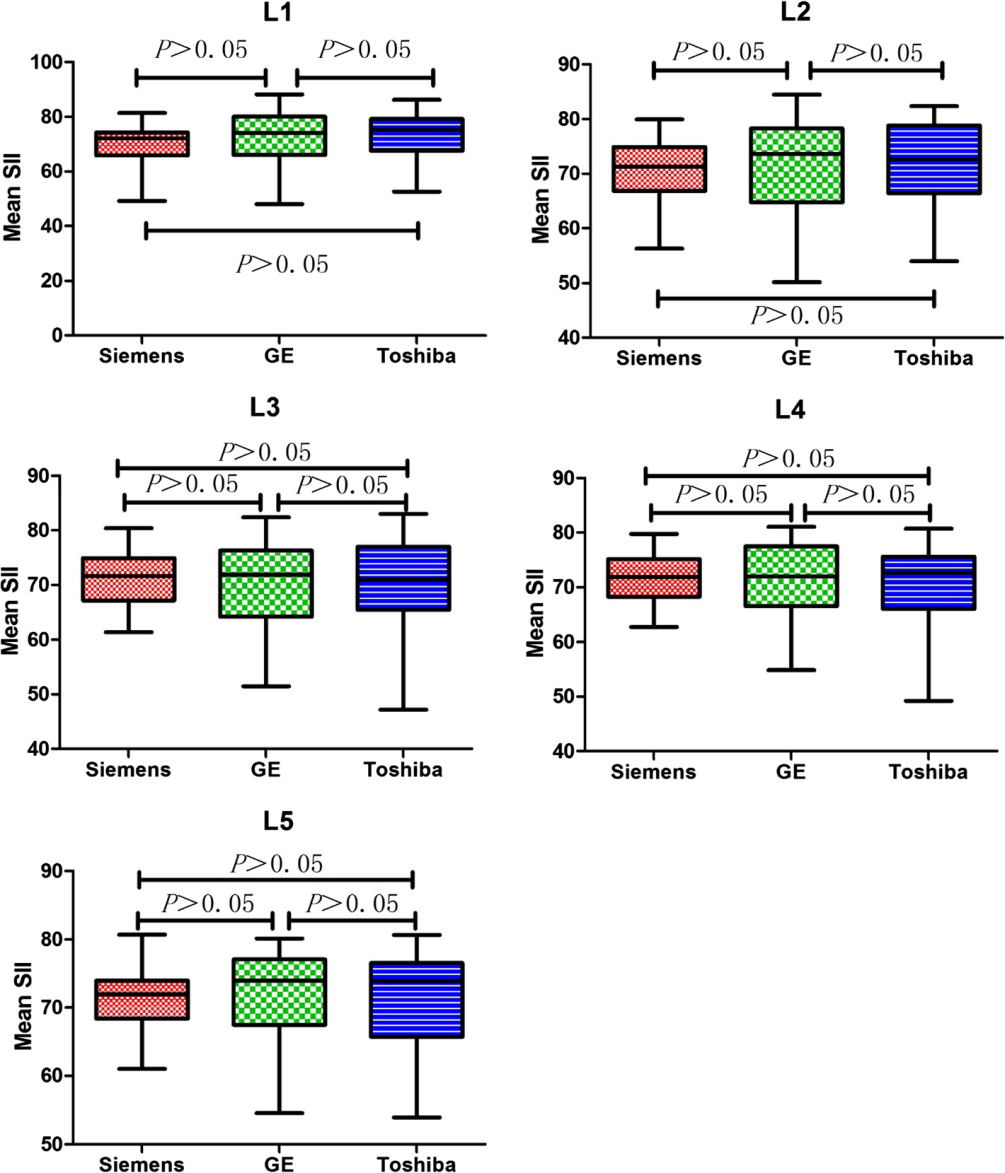
Comparison of mean SII values between different MR systems. In each box, horizontal line is the median, boundaries are 25th and 75th percentiles, and whiskers are lowest and highest data points still within a 1.5 interquartile range of the lower and upper quartiles, respectively. The mean SII values in the same lumbar segment had no significant differences in three different MR vendors

The mean SII values of all lumbar vertebrae at 3.0 T Siemens, 1.5 T GE and 1.5 T Toshiba were 70.9 ± 5.4%, 71.3 ± 5.4%, and 71.5 ± 8.2%, respectively. Table 4 and Fig. 3 summarized the comparison of mean SII values with 3 MR systems in different lumbar segments. From the aspect of different field strength of MR systems, there were no significant differences in mean SII values (3.0 T Siemens vs 1.5 T GE, *p* = 0.337; 3.0 T Siemens vs 1.5 T Toshiba, *p* = 0.561). From the aspect of the same field strength of different vendors (1.5 T GE vs 1.5 T Toshiba), there were no significant differences in mean SIIs (*p* = 0.824). Moreover, the mean SII values in the same lumbar segment had no significant differences among the measurement in the three different MR vendors (all *p* > 0.05, range from 0.26 to 0.95), and the mean SIIs for each MR vendor at different lumbar segments had no significant differences either (all *p* > 0.05, ranging from 0.17 to 0.88).

**Table 4:** Comparison of Mean SII Measurement in Each of the Five Lumbar Segments with Three Vendors in Two Field Strengths

	3.0 T Siemens	1.5 T GE	1.5 T Toshiba	*P* Value^*^
L1	70.124 ± 6.865	72.564 ± 10.121	73.505 ± 8.429	0.257
L2	70.711 ± 5.721	71.156 ± 9.278	71.987 ± 8.255	0.810
L3	71.223 ± 4.939	70.010 ± 8.401	69.917 ± 8.642	0.737
L4	71.344 ± 4.953	71.077 ± 6.925	71.015 ± 6.909	0.930
L5	71.225 ± 4.458	71.601 ± 6.882	71.658 ± 6.822	0.949
*P* Value^+^	0.882	0.171	0.643	

Note. —Data are mean SIIs (%) ± standard deviation

^*^ Comparison of Mean SIIs in the left- right direction

^+^ Comparison of Mean SIIs in the superior- inferior direction

### Comparison of mean SII values in different flip angles

**Fig. 4:**
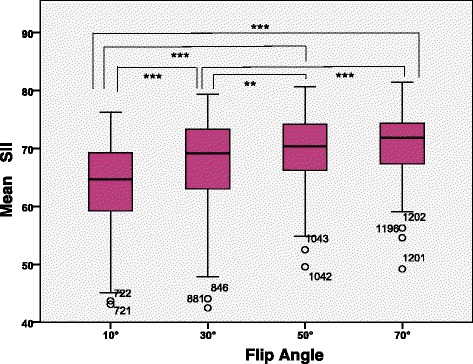
Graph shows SII values for each flip angle at 3.0 T Siemens MR scanner. In each box, horizontal line is the median, boundaries are 25th and 75th percentiles, straight line (bar) on each box is the range of data distribution and empty circles represent outliers (value > 1.5 box length from the 75th and 25th percentile). **: *p* < 0.01; ***: *p* < 0.001

**Fig. 5:**
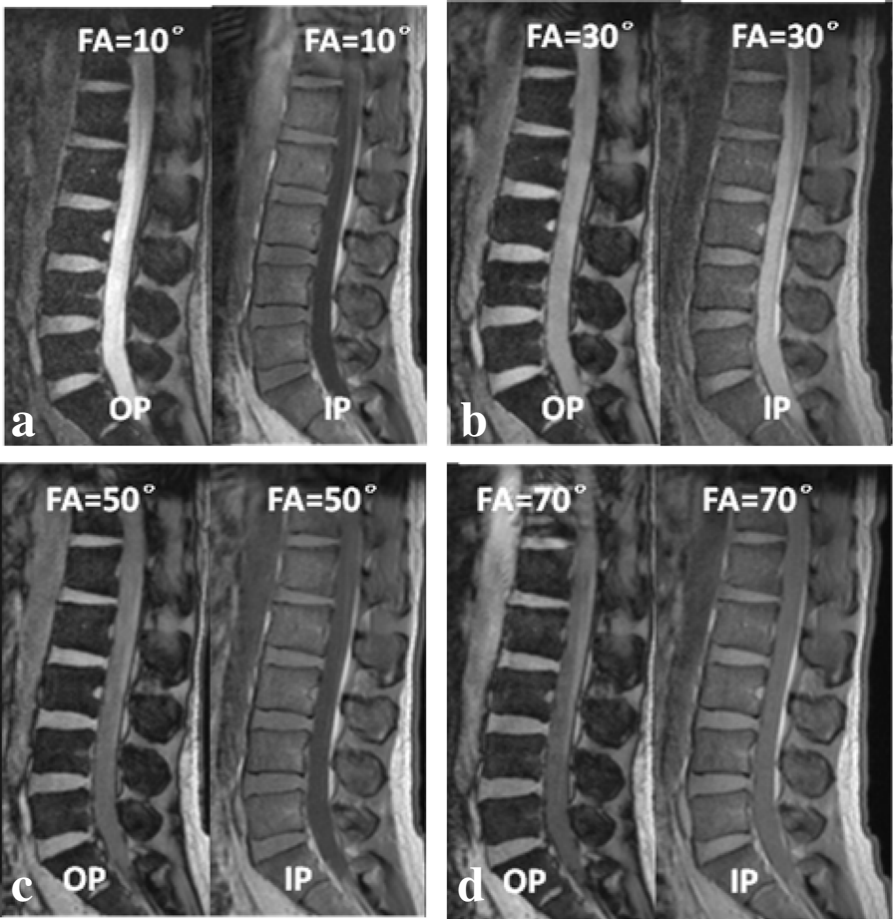
Representative images of chemical shift MR imaging in different flip angles (FA, flip angle). (**a**) in-phase and opposed-phase images acquired when flip angle = 10°; (**b**) in-phase and opposed-phase images acquired when flip angle = 30°; (**c**) in-phase and opposed-phase images acquired when flip angle = 50°; (**d**) in-phase and opposed-phase images acquired when flip angle = 70°

The mean SII of lumbar vertebra at 3.0 T Siemens with different flip angles (flip angle = 10, 30, 50, 70°) was 63.7 ± 7.4%, 67.7 ± 7.5%, 69.7 ± 6.0% and 71.0 ± 5.5%, respectively. The mean SII values showed a tendency of increasing with flip angles, and there were significant differences between different flip angles (*p* < 0.001) although there was no significant difference between the groups of flip angle 50° and 70° (*p* = 0.116) (Fig. 4, 5).

## Discussion

Chemical shift imaging is an useful technology in abdominal imaging by detecting lipid content [1, 3, 22, 26]. It was initially introduced for the assessment of bone marrow in 1985 by Wismer et al. [27], and also has been widely used in musculoskeletal imaging by measuring SII value for evaluation of bone marrow fat content or differentiation benign from malignant lesions [8, 9, 11–14]. But the lack of standardization in data analysis is a major challenge to the widespread and uniform use of chemical shift MR imaging in musculoskeletal imaging when comparing results of different studies. Furthermore, a reliable interpretation of the results contributed by different centers requires comparability of data acquired with MR systems at different institutions, which are often from different vendors and are operated at different field strengths. To the best of our knowledge, there are no previous reports describing the repeatability of SII measurements in different vendors, field strengths and flip angles.

In the present study, inter- and intraobserver correlation coefficients were good when measuring SII values with different MR systems (all ICC > 0.75), which indicates an excellent repeatability. Further study demonstrated that the mutual agreement of three MR systems was satisfying as the mean differences were very small and most of the data points lied within 95% LOA. Considering that imaging parameters are typically optimized for signal-to-noise ratio (SNR) and/or scan time, a given protocol may induce significant T1-weighting bias in the fat fraction estimate [18]. Our study has also shown that the bias was not systematic but depended on specific lumbar segments and on the different MR systems.

The mean SII values measured in our study for different lumbar segments lied within the previously reported range for all three vendors and for both field strength [15], but the mean SII values were slightly higher than the literature reported, which probably resulted from the effects of T1-weighting amplification induced by the high flip angle in our study. For the same field strength of different MR vendors, we did not find a significant difference in mean SII values in any of the evaluated lumbar segments. Furthermore, the agreement of mean SII values in the same field strength was better than different field strengths. All of these pointed to the conclusion that quantitative analysis for lumbar fat content with chemical shift imaging in different MR vendors of the same field strength is comparable.

With the improvements in MR technology, the theoretical advantage of an increased SNR provided by the higher field strength is paralleled by disadvantages and challenges [19, 22]. Moreover, there exists fundamental differences in the MR physics of 3.0- and 1.5-T MR systems. As a result, chemical shift MR imaging at 1.5 T cannot be applied to 3.0 T MR imaging [19]. Thus, the repeatability of different field strengths is necessary to be researched. In this context, we found no significant difference in mean SII values with 1.5- and 3.0-T MR systems, and the repeatability of mean SII values in different field strength were good. Although the literature had reported that there were two factors influencing SI loss on OP images: chemical shift effect and susceptibility effect (i.e. T2* decay), and susceptibility effect occurred due to field strength inhomogeneity which was stronger at 3.0 T than at lower field strength [18, 19, 28], the results in our study indicated that quantitative analysis for lumbar fat content with chemical shift imaging in different field strengths MR systems may be comparable. Sebastian et al. had investigated the SII at 3.0- and 1.5-T MR imaging for prospectively quantitative analysis in a phantom study, and the result was similar to what we obtained.

In theory, the SI loss on OP images should be sensitive to differences in T1 relaxation, except for chemical shift effect and susceptibility effect [19]. With a poor choice of TE, susceptibility artifact on an OP image acquired later than an IP images can occur and may lead to the misinterpretation of a malignant adrenal lesion as a benign adenoma [22]. As a result, there were several studies focusing on effect of echo time in chemical shift MR imaging, but little or none literatures discuss the effects of T1 relaxation, which introduces a dependence on imaging parameters such as flip angle. In our current study, we did find no significant difference between the group of flip angle 50° versus 70° while there were significant differences between other groups. Moreover, the mean SII values increased with the augment of flip angle. The reason may be that a low flip angle could decrease the SNR leading to a bias in measuring SII and smaller estimates, and/or that due to the shorter T1 of fat, a low flip angle would influence full T1 recovery and make the ratio TE/TR changed.

The result of our study indicated that chemical shit MR imaging might be applicable as a biomarker in the lumbar spine, even in multicenter studies combining different vendors and different field strengths. For instance, studies that focus on treatment-induced SII value differences can be performed with any combination of MR systems. Ongoing research could focus on providing correction factors for intervendor or interfiled strength comparisons or further optimizations of SNR to reduce overall test-retest variability.

Our study still have some limitations. Firstly, we included healthy volunteers instead of a phantom for measurements. But we aimed to simulate a clinical environment similar to clinical practice for our analysis. Secondly, we did not evaluate the reproducibility in subjects with pathology to make meaningful comparisons. A large number of patients with different diseases in various vertebrae or organs would be necessary. However, it may be difficult to acquire a large number of patients with the disease who would be willing to undergo three repeated measurements. Thirdly, subjected to the limited conditions, we did not strictly compare mean SII values between different filed strength at the same vendor. A next multicenter study will be performed to refine our research. Lastly, as SIIs measured in chemical shift MR imaging are not quantitative, quantitative MR measurement including T1 and T2 relaxation times, such as a proton density fat fraction (PDFF) should be intended in our further research.

## Conclusion

In conclusion, the signal index (SII) using chemical shift MR imaging may be comparable between MR systems from different vendors and at different field strengths. In addition, high flip angles (50° or 70°) showed better stability for quantitative analysis of lumbar fat content, indicating that high flip angles should be chosen when other parameters are fixed.

## Abbreviations

FA: Flip angle
ICC: Interclass correlation coefficients
IP: In phase
LOA: limits of agreement
MRI: Magnetic resonance imaging
OP: Out of phase
PDFF: Proton density fat fraction
SII: Signal intensity index
SNR: Signal-to-noise ratio
STIR: Short inversion time inversion-recovery
TR: Repetition time
